# Nutritional Status and Other Clinical Variables Are Associated to the Resting Energy Expenditure in Patients With Chronic Kidney Disease: A Validity Study

**DOI:** 10.3389/fnut.2022.881719

**Published:** 2022-05-18

**Authors:** Samuel Ramos-Acevedo, Luis Rodríguez-Gómez, Sonia López-Cisneros, Ailema González-Ortiz, Ángeles Espinosa-Cuevas

**Affiliations:** ^1^Department of Nephrology and Mineral Metabolism, Instituto Nacional de Ciencias Médicas y Nutrición Salvador Zubirán, Mexico City, Mexico; ^2^Programa de Maestría y Doctorado en Ciencias Médicas y Odontológicas y de la Salud, Universidad Nacional Autónoma de México, Mexico City, Mexico; ^3^Translational Research Center, Instituto Nacional de Pediatría, Mexico City, Mexico; ^4^Department of Health Care, Universidad Autónoma Metropolitana, Mexico City, Mexico

**Keywords:** energy requirements, nutritional attention, indirect calorimetry, resting energy expenditure, chronic kidney disease, energy, equation, validity

## Abstract

**Background:**

Estimating energy requirements (ER) is crucial for nutritional attention to chronic kidney disease (CKD) patients. Current guidelines recommend measuring ER with indirect calorimetry (IC) when possible. Due to clinical settings, the use of simple formulas is preferred. Few studies have modeled equations for estimating ER for CKD. Nevertheless, variables of interest such as nutritional status and strength have not been explored in these models. This study aimed to develop and validate a model for estimating REE in patients with CKD stages 3–5, who were not receiving renal replacement therapy (RTT), using clinical variables and comparing it with indirect calorimetry as the gold standard.

**Methods:**

In this study 80 patients with CKD participated. Indirect calorimetry (IC) was performed in all patients. The calorimeter analyzed metabolic measurements every minute for 15 min after autocalibration with barometric pressure, temperature, and humidity. Bioelectrical Impedance Analysis (BIA) was performed. Fat-free mass (FFM) was registered among other bioelectrical components. Handgrip strength (HGS) was evaluated and an average of 3 repetitions was recorded. Nutritional status was assessed with the subjective global assessment (SGA). Patients categorized as B or C were then considered as having malnutrition.

**Results:**

We analyzed 71 patients and 3 models were generated. Model 1a included FFM; Model 2a included weight; Model 3c included handgrip strength (HGS). All other variables were stepwise, computer-selected with a *p* < 0.01 significance level; Malnutrition was consistently associated with ER among other clinical variables in all models (*p* < 0.05). The model that included BIA-FFM had *R*^2^_*adjusted*_ = 0.46, while the model that included weight (Kg) had an adjusted *R*^2^_*adjusted*_ = 0.44. The models had moderate concordance, LC = 0.60–0.65 with the gold standard, whereas other energy expenditure estimation equations had LC = 0.36 and 0.55 with indirect calorimetry. Using these previously validated equations as a reference, our models had concordance values ranging from 0.66 to 0.80 with them.

**Conclusion:**

Models incorporating nutritional status and other clinical variables such as weight, FFM, comorbidities, gender, and age have a moderate agreement with REE. The agreement between our models and others previously validated for the CKD patient is good; however, the agreement between the latter and IC measurements is moderate. The KDOQI lowest recommendation (25 Kcals/kg body weight) considering the 22% difference with respect to the IC for total energy expenditure rather than for REE.

## Introduction

Estimating energy requirements (ER) is a crucial step in providing nutritional care to any population, especially to those at risk of malnutrition (1). Patients with chronic kidney disease (CKD) undergo a variety of physiological changes that may affect their ER and, thus, their nutritional status (2, 3). Historically, only a few studies have demonstrated a neutral energy balance with patients with CKD consuming approximately 30 kcal/day (4, 5). On the other hand, there is evidence of a lack of agreement between estimates of ER and actual resting energy expenditure (REE), which is frequently lower than estimated (6). Whenever it is possible, current guidelines recommend measuring ER using indirect calorimetry (IC) (7). Due to the complexity of clinical settings, simple formulas, and clinical criteria based on body composition, age, sex, comorbidities, and nutritional status, among others, such as inflammation, are also suggested for assessing ER. However, their quantitative contribution has not been established, and the general recommendation for estimating ER is the simple formula of 25–35 kcal/kg/day (7).

In 2021, two distinct studies (8, 9) published more complex and comprehensive formulas for estimating REE, both of which included body composition, gender, age, and comorbidities as indirect variables in linear regression models. However, none of the other variables suggested in the guidelines were studied. Subjective global assessment (SGA) is a valid tool for assessing nutritional status in patients with CKD (10).

Previous research has shown that SGA has an association with body composition when evaluated with BIA (11). Thus according to Steiber et al. (12) SGA classification may be a useful inclusion in an equation for estimating REE. On the other hand, while fat-free mass (FFM) is the most important variable associated with REE, current evidence suggests that FFM should be considered not only in terms of quantity but also in terms of quality due to the numerous clinical outcomes associated with it (including survival and improved QoL) (13). Thus, handgrip strength (HGS) as an overall functional-quality muscle mass may be of interest for estimating REE and providing better nutritional care.

Additionally, renal patients experience hydration changes and are more prone to overhydration, which has been shown in previous work to alter ER as measured by IC in peritoneal dialysis patients (14). Bioelectrical impedance analysis is not only useful for estimating FFM but also for studying patients’ hydration status. Using vectorial bioimpedance analysis (VIBA), a qualitative analysis presented in figures based on the hydration status of a healthy population, (15) thus this study aimed to develop and validate a model for estimating REE in patients with CKD stages 3–5 who were not receiving renal replacement therapy (RTT) using clinical variables and comparing it with indirect calorimetry as the gold standard. Additionally, we aimed to compare our equation with previously validated equations using CKD as a congruent validation method for estimating REE.

## Materials and Methods

This was a validity study involving 80 patients with CKD, who were seen in the outpatient clinic of our institution. It was conducted in accordance with the established standards of good clinical practice and with the approval of the ethics and the research institutional review boards. Patients with a diagnosis of CKD stages 3–5 without RRT, an eGFR estimated using the CKD-epi equation, and any comorbidity (i.e., Diabetes Mellitus, Hypertension, Glomerulopaties, and others such as Lupus) were included if they consented to participate. Patients who were missing a limb or had a metal plate implanted in their bodies were excluded from the study. Those who agreed to participate in the study attended to the metabolic, body composition, and clinical tests after a minimum of 4 h of fasting.

### Metabolic Analysis

All the patients underwent IC using a CardioCoach VO2 max (Korr Medical Technologies Inc., Salt Lake City, Utah). The patients wore a face mask connected to the calorimeter, and a computer recorded variables such as VO2, CO2, FEO2, FECO2, and heart rate. After autocalibration with barometric pressure, temperature, and humidity, as well as the respiration stabilization phase, the calorimeter analyzed the aforementioned variables in a computer interphase every minute for 15 min. The patients were placed in a supine position for 5 min prior to the start of the test. The calorimeter software was programmed with the user’s weight, age, height, and gender. We considered data from the software from patients with stable calorimetry analysis defined as a respiratory coefficient between the physiological ranges [(QR) = 0.68–1.2] or having at least 1 period with less than 10% in coefficient variation (1).

### Body Composition Tests

A Quadscan 4000 (Bodystat, Isle of Man), was used to conduct a bioelectrical impedance analysis (BIA). Following the IC, the patients were positioned supine. At 50 kHz, the reactance (Xc), resistance (R), and phase angle (PA) were measured and standardized by height. The FFM was determined in addition to other bioelectrical parameters such as phase angle.

### Clinical Examinations

A hand dynamometer (Takei Scientific Instruments Co., Japan) was used to conduct strength tests. The patients were instructed and encouraged to squeeze as hard as possible and maintain their strength for 3–4 s. The average of three repetitions with the dominant arm was recorded.

Nutritional status was evaluated in 5 domains using SGA: (1) weight changes; (2) dietary changes; (3) functional capacity changes (i.e., daily activities); (4) muscle and fat storage changes; and (5) presence of edema or ascites. This tool categorizes a person’s nutritional status into three groups: (A) appropriate nutrition; (B) mild to moderate malnutrition; and (C) severe malnutrition. Malnutrition was then considered to be present in patients categorized into the B or C group.

Recent laboratory data (within 1 month) were obtained from the medical record, including electrolytes, uremic waste products, and serum creatinine. The CKD-epi equation was used to calculate the estimated glomerular filtration rate (eGFR).

In order to determine construct validity as secondary objective, we compared previously validated formulas to our data and vice versa. The following formulas were analyzed:

• REE: 668 + (17.1*FFM-BIA kg)—(2.7*age years) – (92.7*sex) + (1.3*eGFR_*CKD*–*epi*_) – (152.3 if having diabetes mellitus); sex: 0 = woman, 1 = male (8)
• REE: 854.5 + (7.4*weight kg) + (179.3*Sex) – (3.3*age years) + (2.1*eGFR_*CKD*–*epi*_) + (25.6 if having diabetes mellitus); sex: 0 = woman, 1 = male (8)
• REE: 645.5 + (–4.7*age years) + (106*sex) + (13.1*weight kg) + (–51.6 if having DM) (9)
• REE: 25 kcal*actual weight (kg) (7)
• REE: Woman: 665 + (9.56 × weight Kg) + (1.85 × height cm) – (4.68 × edad)
• REE: Male: 66.5 + (13.75 × weight Kg) + (5 × height cm) – (6.78 × edad)

### Sample Size

The sample size was determined using the Freeman equation (16), which states that ten people should be included for every K + 1 variable in a regression analysis, for both, qualitative and quantitative analysis [*n* = 10 (K + 1), where K is the model’s number of variables]. We considered five variables to include in the model, which resulted in a sample size requirement of 60 people plus 20% of possible missing data, or 72 patients.

### Statistical Analysis

The descriptive statistics were consistent with the variable distribution using the Kolmogorov-Smirnoff test. This study aimed to evaluate an *a priori* model proposed by an expert panel. We used a modified Delphi methodology to determine the content validity of a hypothesized renal-specific REE equation (data not published). Discussion took place during three rounds where a moderator gave feedback using all the comments and suggestions provided by the panel. Accordance was set as the case when 80% of the board agreed with the statement. It was agreed to include age, sex, and fat-free mass and evaluate SGA and HGS. The Delphi panel assessed the plausibility of the variables to be included in the model.

Correlations between quantitative and qualitative data were determined. We conducted a linear regression analysis with REE determined by calorimetry (IC-REE) as the dependent variable. The model was fitted stepwise, with a *p*-value of 0.1 for variable inclusion. Beta coefficients were calculated and used to predict fitted values, while standardized coefficients provided additional information. As suggested, the regression assumptions were analyzed. The model-fitted values were analyzed using the intraclass correlation coefficient (ICC), with IC measurements serving as the gold standard (convergent criterion validity). The concordance analysis was evaluated with Bland-Altman (BA) graphs accompanied by the Lins concordance coefficient (LCC). The BA method determined the differences between the IC-REE and fitted values. The data was single paired measurements (fitted values vs. gold standard). Acceptable limits of agreement were considered for 300 Kcal. The BA assumptions were analyzed with the normality of the differences from methods with q-q graphs and the Kolmogorov-Smirnoff test. At the same time, homoscedasticity was evaluated qualitatively with residuals graphs post-regressing the methods’ means and their differences. If the assumptions were not satisfied, logarithmic transformation was considered. Proportional bias was analyzed using a regression line in the Bland-Altman figure. Any deviation from the zero line, indicating a linear trend, was considered proportional bias (where the variability of differences between methods increases as the magnitude of the measurement increase, or vice-versa). In such case, BA figures were presented as percentage differences with bias, and LoA’s based on the regression analysis (17–19). As for the secondary objective, the same statistical method was applied, but comparing our models with other authors’ models. STATA 15.1 (College Station, Texas) was used to analyze the data.

## Results

Eighty patients participated in the study, with 71 having an accurate IC measurement. Table 1 contains descriptive statistics. Females constituted the majority of the patients, with a median eGFR of 33 (16–47) ml/min/1.73 m^2^. Additionally, 16.9% of the study population identified by SGA had mild to severe malnutrition. The patients had a higher prevalence of hypertension than diabetes. The population had a mean BMI of 26.48 ± 4.92 kg per square meter, an impedance phase angle (PA) of 5.66 ± 1.14, and an HGS of 25 ± 9.6 kg per strength. By IC, the mean ER was 1386.23 ± 393.5 kcal/day, with a median respiratory quotient of 0.67 (0.64–0.69).

**TABLE 1:** General characteristics of the study population.

Variable	Value *n* = 71
**General characteristics**	
Age (years)	53 (32–61)
eGFR (ml/min)	33 (16–47)
CKD stage *n* (%)	
3	38 (53.5)
4	18 (25.4)
5	15 (21.1)
Sex *n* (%) (female)	38 (53.52)
Diabetes mellitus *n* (%)	23(32.39)
Hypertension *n* (%)	31 (43.66)
**Laboratory tests**	
Glucose (mg/dl)	86 (81–102)
BUN (mg/dl)	38.7 (28.2–49.6)
Urea (mg/dl)	80.04 (60.35–101.65)
Creatinine (mg/dl)	2.14 (1.59–3.52)
P (mg/dl)	3.88 (3.47–4.25)
K (mg/dl)	4.64 (4.31–4.92)
Na (moll/l)	139 (138–141)
**Body composition and nutritional measurements**	
Weight (kg)	67.4 (54.5–79.2)
BMI (kg/m^2^)	26.48 ± 4.92
Lean mass (kg)	47.07 ± 12.2
Fat mass (kg)	19.65 (14.9–26.6)
R/H (Ω/m)	330 (276–406)
R (Ω)	538.3 ± 114.8
Xc/H (Ω/m)	33.84 ± 10.64
Xc (Ω)	54 ± 16.7
PA°	5.66 ± 1.14
Subjective global assessment *n* (%)	
Normal	59 (83.1)
Mild to moderate	11 (15.5)
Severe	1 (1.4)
HGS right (kg/Strength)	25.02 ± 9.61
**Indirect calorimetry parameters**	
Energy kcal	1386.23 ± 393.48
Respiratory quotient	0.67 (0.64–0.69)
VO_2_	201.24 ± 56.7
VCO_2_	134.06 ± 37.08

*eGFR, estimated glomerular filtration rate; Na, sodium; K, potassium; BUN, blood urea nitrogen; P, phosphorus; R/H, resistance adjusted from height; Xc/H, reactance adjusted from height; PA, phase angle HGS, handgrip strength; VO_2_, oxygen rate; VCO_2_, carbon dioxide production; BMI, body mass index. Data are expressed as mean and ± SD and median and P25-P75.*

**FIGURE 1:**
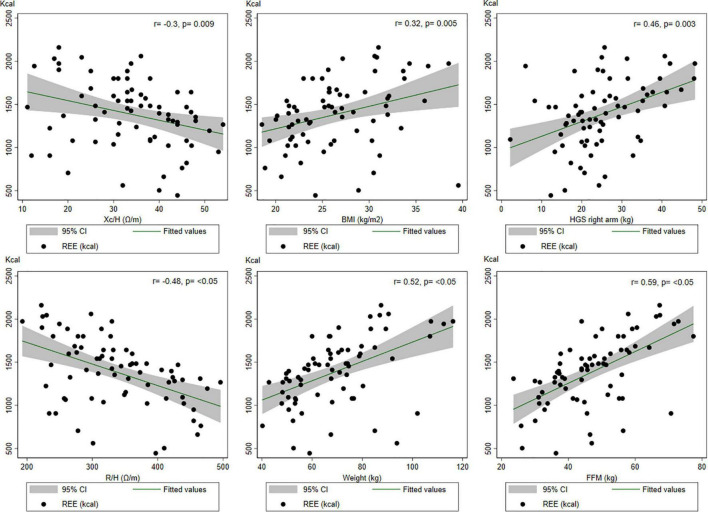
Correlations between some variables of interest and the indirect calorimetry measurements.

Figure 1 illustrates the correlations. The HGS, weight, and FFM all correlated positively with energy measured *via* IC, with the strongest correlation being FFM (*r* = 0.59; *p* < 0.01). Impedance components such as R and Xc, on the other hand, were negatively correlated with IC measurements. The correlation coefficient between the eGFR CKD-EPI and the IC measurement was 0.19, *p* = 0.106. The correlation coefficients for categorical and quantitative data were 0.39 for hypertension and 0.11 for diabetes mellitus and IC measurements, respectively, whereas malnutrition was negatively correlated with the ER at *r* = −0.4.

### Linear Regression

The regression models are presented in Table 2, with Model 2a incorporating BIA-FFM, Model 2b substituting weight (kg) for FFM, and Model 3c replacing weight for dominant HGS. All other variables were computed-selected stepwise with a *p* = 0.01 significance level. The model that included BIA-FFM had an adjusted *R*^2^ of 0.46, while the model that included weight (kg) had an adjusted *R*^2^ of 0.44.

**TABLE 2:** Linear regression for indirect calorimetry measurements (Fat-Free Mass) (Weight), and (Handgrip strength).

Variable	Standardized beta	Coefficient beta	IC 95%	*P*-value
**A**				

Fat free mass (BIA-Kg)	0.57	18.58	12.89–24.27	0.000
Nutritional status (SGA B or C)	−0.31	−325.55	−508.56 to −142.5	0.001
Hypertension (diagnosis)	0.21	167.31	28.42 – 306.21	0.019
Constant	−	489.2	212.1–766.31	0.001

**B**				

Weight (Kg)	0.37	8.49	3.78–13.19	0.001
Nutritional status (SGA B or C)	−0.25	−265.34	−471.79 to −58.89	0.013
Sex (male)	0.24	195.24	36.01–354.46	0.017
Hypertension	0.27	212.93	64.62–361.24	0.006
Age (years)	−0.24	−6.05	−10.93 to −1.16	0.016
Constant	−	959.35	601.6–1317.1	0.000

**C**				

Hand grip strength (Kg)	−0.04	−1.82	−14.1	10.5
Hypertension	0.32	258.5	103.8–413.2	0.001
Age (years)	−0.20	−5.1	−10.4–0.39	0.069
Height (cm)	0.29	5.2	0.94–22	0.033
Nutritional status (SGA B or C)	−0.25	−279.7	−521 to −38.3	0.001
Sex (male)	0.32	190	−37.1 – 417.21	0.100
Constant	−	−323.4	−1914.6 – 1268	0.686

*A: R^2^_adjusted_ = 0.46; R^2^ = 0.48; P_model_ = 0.000. B: R^2^_adjusted_ = 0.44. R^2^ = 0.48; P_model_ = 0.000. C: R^2^_adjusted_ = 0.43; R^2^ = 0.37; P_model_ = 0.000. BIA, bioelectric impedance analysis; SGA, subjective global assessment.*

### Validity: Concordance and Consistency

**FIGURE 2:**
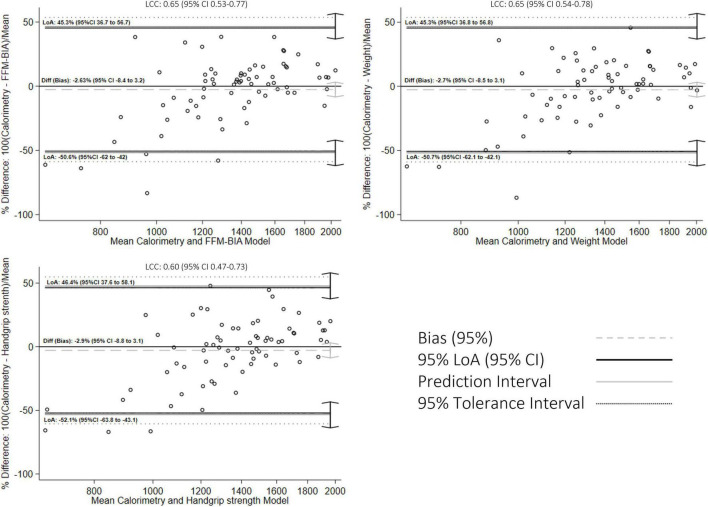
Bland-Altman graph for concordance analysis between fitted values and indirect calorimetry measurements.

**FIGURE 3:**
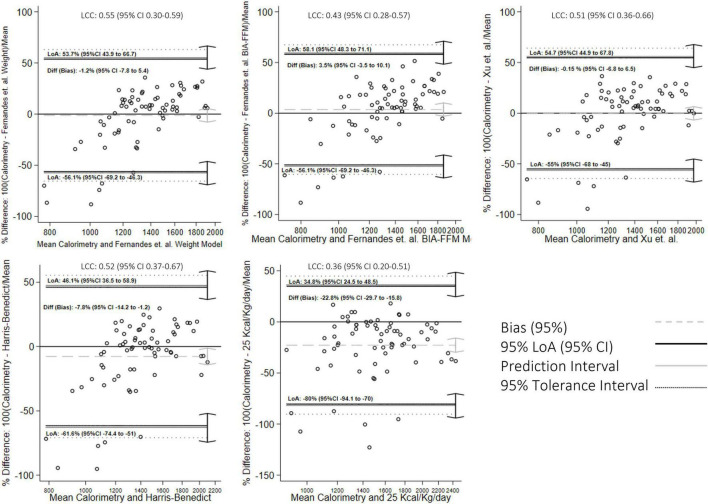
Bland-Altman graph for concordance analysis between previously validated equations with indirect calorimetry measurements.

**FIGURE 4:**
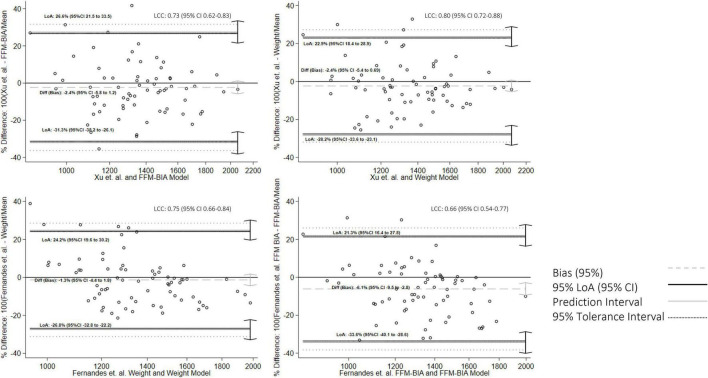
Bland-Altman graph for concordance analysis between fitted values and predictions from previously validated equations.

Additional concordance analysis is presented in Figures 2–4. The BA figures are shown in Figures 2–4. Some models (de Oliveira Fernandes et al. weight equation, Xu et al. equation, Harris-Benedict, and the KDOQI 25 Kcal/Kg/day) did not have parametric distribution on their differences with the gold standard and were log-transformed. Nevertheless, the normality did not improve. We decided to continue the analysis regardless of this limitation with the nature of the variable. All equations showed proportional bias and are presented in mean percentage difference. Most of the equations, except the Harris-Benedict and the KDOQI 25 Kcal/Kg/day, had non-significant percent mean differences, although all models had more significant than expected LoA. Our models had LCC values between 0.60 and 0.65 (Figure 2), whereas other energy expenditure estimation equations had LCC values between 0.36 and 0.55 (Figure 3 and Table 3). Using these previously validated equations as a reference, our models had LCC values ranging from 0.66 to 0.80 (Table 4 and Figure 4), and the percent mean difference was no greater than 10% between them. The only significant percent mean difference was observed in de Oliveira Fernandes et al. BIA-FFM equation and our model, including BIA-FFM.

**TABLE 3:** Estimated calories and intraclass correlation coefficients between our models, and other authors, with indirect calorimetry measurements.

Equation *n* = 71	Estimated calories (REE)	ICC (95% CI)	LCC (95 % CI)
Indirect calorimetry	1,386 ± 393	ND	ND
BIA-FFM (Kg)	1,386 ± 275	0.66 (0.50–0.77)	0.65 (0.53–0.77)
Weight (Kg)	1,386 ± 275	0.66 (0.50–0.77)	0.65 (0.54–0.78)
Handgrip strength (Kg/strength)	1,386 ± 258	0.60 (0.43–0.73)	0.60 (0.47–0.73)
Xu et al, weight (Kg)	1,350 ± 255	0.51 (0.32–0.66)	0.51 (0.36–0.66)
De Oliveira et al (weight)	1,356 ± 204	0.45 (0.25–0.62)	0.55 (0.30–0.59)
De Oliveira et al (BIA-FFM)	1,293 ± 204	0.43(0.22–0.60)	0.43 (0.28–0.57)
Harris-Benedict	1,457 ± 270	0.52 (0.33–0.67)	0.52 (0.37–0.67)
KDOQI guidelines (25 Kcal/kg)	1,726 ± 426	0.36 (0.01–0.60)	0.36 (0.20–0.51)

*BIA-FFM, fat-free mass determined with bioelectrical impedance; ICC, intraclass correlation coeffficient; LCC, lins concordance coefficient; REE, resting energy expenditure.*

**TABLE 4:** The intraclass correlation coefficient between previously validated equations, as a standard reference, and our models (*n* = 71).

Equations in comparison	ICC (CI 95%)	LCC (CI 95%)
BIA-FFM vs. de Oliveira Fernandes et al. BIA-FFM model	0.66 (0.43–0.79)	0.66 (0.54–0.77)
Weight vs. de Oliveira Fernandes et al. weight model	0.76 (0.64–0.84)	0.75 (0.66–0.84)
HGS vs. de Oliveira Fernandes et al. weight model	0.65 (0.49–0.76)	0.65 (0.52–0.78)
BIA-FFM vs. Xu et al. model	0.73 (0.60–0.82)	0.73 (0.62–0.83)
Weight vs. Xu et al. model	0.80 (0.70–0.87)	0.80 (0.72–0.88)
HGS vs. Xu et al. model	0.63 (0.46–0.75)	0.62 (0.48–0.76)

*ICC, Intraclass Correlation Coefficient; LCC, Lins Concordance Coefficient; BIA-FFM, Fat-free mass determined with bioelectrical impedance; HGS, Handgrip strength.*

The ICC is presented in Table 4 to analyze consistency between our models and previously validated equations. In comparison to IC measurements, the ICC values for the models with BIA-FFM and weight were 0.66 (0.50–0.77) for both equations. The ICC values for previously validated equations were between 0.5 and 0.6 compared to our IC values (Table 4).

## Discussion

Estimating energy requirements is critical during the nutritional care process since it establishes a portion of the goals for treating the patient based on clinical evidence and critical thinking (20). Specifically for kidney patients, providing an appropriate and individualized nutritional treatment should consider the risk that this population has of developing malnutrition and attempt to avoid adverse outcomes associated with this phenomenon, even in the early stages of the disease (21). Today, various reference guides recommend estimating energy using the reference standard (CI) (1, 7, 22). However, since this method is not widely available, having validated energy estimation equations based on the reference standard is critical. We set out to develop and validate a model for estimating REE of kidney patients in stages 3–5 without RRT and compare our model’s consistency and concordance with those developed recently and some others that are frequently used, such as Harris-Benedict and KDOQI Guidelines (23). We can emphasize the relationship certain variables have with REE, the most significant variable being the FFM determined by BIA (8, 9, 23, 24). These findings are consistent with previous research on body composition and REE, which indicates that, in addition to muscle mass, weight as the sum of all body composition compartments also has a moderate correlation. Around 80% of a person’s REE is determined by body size, with lean body mass having the highest correlation.

According to de Oliveira Fernandes et al.’s model (8), weight in kg accounts for 21% of the variance in REE, and the addition of sex, age, kidney function, and diagnosis of diabetes increases the variance explained to up to 42% of REE. On the other hand, Xu et al. (9) demonstrated that the same model could account for 77% of REE variance with slightly larger sample size. Besides, de Oliveira Fernandes et al. (8) showed that FFM alone could account for between 33 and 36% of REE variability, depending on whether it is estimated using anthropometric measurements or bioelectrical impedance. It should be noted that many of the prediction equations for BIA-FFM used by the analyzers were developed in healthy populations, casting doubt on their validity for use in CKD patients. This is one of the primary reasons for encouraging validation studies of specific clinical tools for each of the pathological entities.

In our models, the weight and FFM estimated by BIA, combined with other variables, account for slightly less than 50% of the variance in REE. These additional variables include nutritional status, a diagnosis of hypertension, sex, and age. Interestingly, despite a modest correlation with REE (*r* = 0.46), The HGS is irrelevant for developing a predictive model. This could be because when other variables are considered, it loses significance in terms of REE. However, given the biological plausibility and proposal in the Delphi consensus described previously and the emphasis placed on evaluating muscle mass and its quality and strength, it is necessary to assess this possibility. Muscle strength loss has been linked to an increased risk of falls, loss of autonomy, and ultimately hospitalization and death, beginning as early as 30 years (25).

Although the guidelines suggest considering nutritional status as a variable when determining how many calories to indicate to the patient to avoid malnutrition, no specific recommendation is made (7). It is interesting to note that nutritional status, as determined by SGA, is a variable that remains statistically significant in all proposed models, lowering REE in the presence of malnutrition (B or C, as determined by SGA). This can be explained by the possible loss of muscle mass that people with this nutritional characteristic may experience; however, the evidence is changing since, while the phenomenon is similar in people with heart failure, the energy demands of people with malnutrition are typically increased in cancer patients (26).

It has been demonstrated that patients who do not receive dialysis may have lower REE levels comparable to healthy people (27, 28). It is suggested that comorbidities generally increase REE. While the diagnosis of diabetes mellitus does not affect the REE of our population, it is worth noting that only 32% of our patients had this diagnosis, compared to 43% who had hypertension, which contributed to the models having a standardized value greater than 0.20. Other proposed models incorporate DM into the equations (8, 9), increasing or decreasing the REE associated with the diagnosis, depending on the variables it interacts within the model. Various comorbidities may play a significant role in modifying REE in the CKD population i.e., catabolic conditions, poorly controlled diabetes, metabolic syndrome, and hyperparathyroidism are all included in these variables (2, 3, 7, 29, 30).

Age and gender are considered to be standard variables to consider when discussing REE (7). Since the beginning of human metabolism research, a strong correlation between these two variables and REE has been demonstrated, and this appears to be the case in renal patients. With increasing age, REE decreases, and it appears as though men require slightly more energy than women. When FFM is omitted from our models, gender, and age play a role.

The simplest method for calculating a person’s energy requirements is to use estimation equations. The validity of the classic formulas is debatable, and the bias they may contain has been documented in several studies, with more than half of the population studied being over—or underestimated (6, 14). In our study, concordance between different models (including those created in this study) is moderate, despite the low mean differences that we found. The LoA are especially wide in those with lower REE in terms of means of methods, and percent means differences reached up to 50% in some equations in comparison with the gold standard. This may be in relation with the sample size, which was calculated for the modeling instead for the concordance and the BA analysis.

Interestingly, using previously validated equations as a reference standard, our models have a moderate to good ICC, particularly when compared to Xiao et al. model (ICC 0.80) (0.70–0.87). The concordance improved, having narrower LoA, however, when their models are applied to our population’s reference standard (CI), the concordance is moderate. These findings are consistent with our observations about the agreement of other equations, such as Harris-Benedict. This may indicate that, while the equations are similar, the estimates vary among populations.

On the other hand, it is essential to note that the most significant difference we found is with the 25 Kcal/Kg/day of the KDOQI guidelines (7). However, we must mention that the 22% difference found could mean the complementary calories to estimate GET instead of GER.

Although there is no acceptable range between calories estimated by prediction equations and calorimetry measurement, 10% variations are considered clinically significant. It is essential to mention that most of the formulas proposed by our team or other authors meet this characteristic in our population assessed with the mean percentage difference, nevertheless LoA are greater than 20% in all cases. The BA figures showed a bias toward overestimating the REE, for that measured by the IC, in people with averages < 1,200 Kcal, nevertheless great percentage of the population is within the LoA when the REE is > 1,200 Kcal.

Certain characteristics of kidney patients (changes in hydration and the bias this may represent in body weight, decreases in GFR *per se*, uremia, anorexia, inflammation, and insufficient physical activity) complicate studies on this subject. These variables are also proposed for consideration in the guidelines for estimating REE, nevertheless some of them are not feasible to in clinical settings (i.e., inflammation studied with PCR).

This study, has some limitations, including the difficulty of determining the external validity of our models due to the lack of an opportunity to apply the equations to a different sample from the one used to create the models. Another significant limitation of this research is the sample size. While we believe that the calculation used to create the regression model is sufficient, it is insufficient to perform finer stratifications or sub-analyses in terms of statistical power. Additionally, since hydration status was not explored in this study, weight may be skewed; however, other authors have proposed weight as a critical variable for measuring REE. It’s also worth noting that the purpose of this study was to quantify REE, which means that exercise and physical activity were excluded by definition.

On the other hand, among the most significant strengths of our work, we can point out that it is one of the few that has been tasked with validating an energy estimation equation for CKD patients and that it also incorporates little-explored variables such as nutritional status and HGS, as suggested in the guidelines and expert consensus prior to this study (under review). Additionally, the work incorporates the models proposed to examine the concordance of their results in our population, indicating that, while the equations are similar, the estimates are not entirely concordant, possibly due to differences in populations. Nevertheless, those weight-based-equations seems to be the more consistent, and concordant and possibly the more valid to estimate REE in CKD patients in stages 3–5.

## Conclusion

We can conclude that models incorporating nutritional status and other clinical variables such as weight, FFM, comorbidities, gender, and age have a moderate degree of agreement with REE measurements obtained *via* IC and have moderate validity. The concordance between our models and others previously validated for the CKD patient is high; however, the agreement between the latter and IC measurements is moderate, noticing that the concordance and dispersion of the data are significantly biased in those with energy expenditure below 1,200 Kcal, nevertheless for values > 1,200 kcal, the patients were in between the LoA. to the results of this study, we suggest the use of formulas based on body weight, as well as the use of the KDOQI lowest recommendation (25 Kcals/kg body weight) considering the 22% difference with respect to the IC for total energy expenditure rather than for REE.

## Data Availability Statement

The raw data supporting the conclusions of this article will be made available by the authors, without undue reservation.

## Ethics Statement

The studies involving human participants were reviewed and approved by the Comité de Etica e Investigación del Instituto Nacional de Ciencias Médicas y Nutrición Salvaldor Zubirán No. 3045. The patients/participants provided their written informed consent to participate in this study.

## Author Contributions

SR-A and LR-G participated in the research generation, carrying, collection, analysis of the data, and in writing the article. SL-C and AG-O participated in the interpretation of the data and revision of the manuscript. ÁE-C participated in study conception and design, revision, analysis of the data, writing the manuscript, revision, and approval of the final version of the manuscript. All authors contributed to the article and approved the submitted version.

## Conflict of Interest

The authors declare that the research was conducted in the absence of any commercial or financial relationships that could be construed as a potential conflict of interest.

## Publisher’s Note

All claims expressed in this article are solely those of the authors and do not necessarily represent those of their affiliated organizations, or those of the publisher, the editors and the reviewers. Any product that may be evaluated in this article, or claim that may be made by its manufacturer, is not guaranteed or endorsed by the publisher.
